# Assay of hemoglobin A_1c_ using lectin from *Aleuria aurantia*

**DOI:** 10.1186/s13568-016-0288-7

**Published:** 2016-11-22

**Authors:** Masato Kabata, Erina Hase, Kouta Kimura, Yuka Kobayashi, Yasushi Ueno, Kazuaki Yoshimune

**Affiliations:** 1 Department of Applied Molecular Chemistry, Graduate School of Industrial Technology, Nihon University, 1-2-1, Izumichou, Narashino, Chiba 275-8575 Japan; 2 Department of Applied Molecular Chemistry, College of Industrial Technology, Nihon University, 1-2-1, Izumichou, Narashino, Chiba 275-8575 Japan; 3 Product Development Laboratory, J-oil Mills Inc., 11, Kagetoricho Totsuka-ku, Yokohama, Kanagawa 245-0064 Japan; 4 Nacalai tesque Inc., Kyoto, Japan

**Keywords:** Lectin, *Aleuria aurantia*, Hemoglobin A_1c_, Lectin-based enzyme-linked immunosorbent assay

## Abstract

Hemoglobin A_1c_ (HbA_1c_) has an *N*-terminal fructosyl valine on the β-chain, and this modification is caused by the non-enzymatic glycosylation of hemoglobin (Hb). The relative concentration ratio of HbA_1c_ to total Hb is an important biomarker for the diagnosis of diabetes. HbA_1c_-binding lectins were screened from 29 sources of lectin, and the lectin from *Aleuria aurantia* (AAL) was revealed to have higher affinity to HbA_1c_ than to Hb. The concentration of HbA_1c_ was determined by lectin-based enzyme-linked immunosorbent assay (ELISA) using the AAL lectin. Higher reproducibility of the assay was observed at 4 °C than at 25 and 37 °C. This observation is consistent with the known temperature-dependent behavior of lectins. Preincubation of HbA_1c_ with an anti-HbA_1c_ antibody inhibited the binding, suggesting that AAL binds to the *N*-terminal fructosyl valine epitope of HbA_1c_. Higher inhibitory effect was observed for 10 mM d-fructose than for the same concentrations of l-fucose, d-fucose, or d-glucose.

## Introduction

The number of people with diabetes is increasing globally, especially in developing countries, with over 346 million people diagnosed worldwide (Little and Rohlfing 2013). The major hallmark of diabetes is high glucose levels in the blood. However, the concentration of glucose in the blood is not reliable for the diagnosis of diabetes because of fluctuations in these concentrations throughout the day. The relative concentration ratio of hemoglobin A_1c_ (HbA_1c_) to hemoglobin (Hb) is a reliable biomarker for the diagnosis and prognosis of diabetes (Little and Rohlfing 2013). HbA_1c_ is produced by a non-enzymatic reaction between glucose (McDonald et al. 1978) and the *N*-terminal valine of the β-chain of hemoglobin in red blood cells. The reaction proceeds via a Schiff base adduct, followed by the Amadori rearrangement to form a stable fructosyl valine, which exists almost exclusively in the pyranose form (Mortensen and Christophersen 1982). Since the half-life of red blood cells is approximately 2 months, the concentration of HbA_1c_ represents the blood glucose levels over the past 2–3 months. Since the first quantitative assay of HbA_1c_ (Trivelli et al. 1971), the assay method has been improved by a rapid automated assay (Klenk et al. 1982) and an automated immunoassay (Fiore et al. 1988), and the enzymatic assay using fructosyl amino acid oxidase (Sakurabayashi et al. 2003). Conventional HbA_1c_ assay methods available to clinical laboratories are based on the chromatographic method (Imagawa et al. 1984), the latex-enhanced immunoturbidimetric method (Holownia et al. 1997), or the enzymatic method using fructosyl-valine oxidase (Hirokawa et al. 2004). The chromatographic method, involving the use of HPLC for the separation of HbA_1c_ and Hb, is rather expensive. The latex-enhanced immunoturbidimetric method monitors increase in solution turbidity, which is caused by the interactions between HbA_1c_ and the antibodies against fructosyl-valine of HbA_1c_ that are coated on the surface of latex beads. In this method, HbA_1c_ is denatured before the assay so that the *N*-terminal fructosyl valine is exposed (Holownia et al. 1997). The enzymatic method uses fructosyl-valine oxidase for oxidation of the glycated *N*-terminal amino group of the partially digested HbA_1c_ to produce hydrogen peroxide, which is subsequently used for color development.

Lectins are carbohydrate-binding proteins produced by many organisms including fungi, animals, plants, and bacteria. Binding affinity of some lectins to glycoprotein increases at lower temperatures (Hayes and Goldstein 1975; Ebisu et al. 1978; Damian et al. 2005). It is hypothesized that water molecules contribute to the binding between sugar and lectin, and tend to be displaced at higher temperatures (Damian et al. 2005). Fungal lectin from *Aleuria aurantia* (AAL) is a fucose binding lectin, and the binding is inhibited by l-fucose, but not by d-fucose. AAL differs from other fucose-binding lectins by having a broad affinity towards l-fucose-containing saccharides (Olausson et al. 2008). AAL can be used for the assay of serum fucosylated haptoglobin for diagnosis of hepatocellular carcinoma (Kondo et al. 1995). AAL also binds to d-arabinose, which lacks the C-6 methyl group of l-fucose (Fukumori et al. 1990), although the binding affinity to it is 30 times weaker than that to l-fucose (Fujihashi et al. 2003). Recombinant AAL can be overproduced in *Escherichia coli* (Fukumori et al. 1990), and the overproduced AAL is stable after incubation at 55 °C for 10 min (Amano et al. 2003). AAL is composed of two identical subunits of approximately 33 kDa, and each subunit has its sixfold β-propeller structure with five l-fucose-binding sites to bind to the α- or β-anomer form of l-fucose (Fujihashi et al. 2003; Wimmerova et al. 2003).

This study revealed an interaction between AAL and HbA_1c_ using a lectin-based ELISA method. This finding can be applied to develop an HbA_1c_ assay for the diagnosis of diabetes. AAL offers several advantages for use in an HbA_1c_ assay, such as its thermostability and the lower cost of production than that of antibody- or enzyme-based assays, which are the currently used methods.

## Materials and methods

### Materials

All biotinylated lectins described in this report were obtained from J-oil Mills, Inc., Tokyo, Japan. ELISA plates (half area 96 well, flat bottom) were purchased from Greiner Bio-One, Frickenhausen, Germany. Human hemoglobin was obtained from Sigma-Aldrich (St. Louis, MO, USA). Highly purified HbA_1c_ was purchased from BBI Solutions (Cardiff, UK). Monoclonal antibody against HbA_1c_ was purchased from Abnova Corp. (Taipei, Taiwan). Bovine serum albumin for the blocking of the plates was purchased from Jackson Immuno Research Laboratories (West Grove, PA, USA). All the other reagents were purchased from Wako Pure Chemical Industries (Osaka, Japan), unless otherwise stated.

### Lectin-based ELISA

Interactions between AAL and Hb or HbA_1c_ were assayed by lectin-based ELISA. Hb or HbA_1c_ was denatured by incubation in 1.0 M acetate buffer (pH 5.0) for 30 min at 25 °C. A volume of 25 μl of denatured Hb or HbA_1c_ (150 μg/ml) was added to the ELISA plate, and each well was washed with PBS-T, consisting of PBS (137 mM NaCl, 2.7 mM KCl, 10 mM Na_2_HPO_4_, 2 mM KH_2_PO_4_) and 0.05 % Tween 20, and then blocked by the addition of 1 mg/ml BSA in PBS at 37 °C for 60 min. After washing with PBS-T, the plate was incubated with 25 μl of biotinylated lectin (5 μg/ml) in PBS with 1 mg/ml BSA for 1 h. Biotin labeled-lectins from *Aleuria aurantia* (AAL), *Agaricus bisporus* (ABA), *Amaranthus caudatus* (ACA), *Agrocybe cylindracea* (ACG), *Arachis hypogaea* (PNA), *Bauhinia purpurea* (BPA), *Canavalia ensiformis* (ConA), *Canavalia gladiata* (CGA), *Galanthus elwesii* (GEA), *Glycine max* (SBA), *Dolichos biflorus* (DBA), *Datura stramonium* (DSA), *Erythrina cristagalli* (ECA), *Galanthus elwesii* (GEA), *Hippeatrum hybrid* (HHA), *Hygrophorus russula* (HRL), *Lens culinaris* (LCA), *Lotus tetragonolobus* (Lotus), *Maackia amurensis* (MAM), *Maclura pomifera* (MPA), *Phaseolus vulgaris* (PHA-E4 and PHA-L4), *Pholiota squarrosa* (PhoSL), *Psophocarpus tetragonolobus* (PTA-I), *Ricinus communis* (RCA120), *Sambucus sieboldiana* (SSA), *Triticum vulgaris* (WGA), *Tulipa gesneriana* (TxLc-I), *Ulex europaeus* (UEA-I), and *Vicia villosa* (VVA-G) were screened. After washing with PBS-T, 25 μl of high sensitivity streptavidin-HRP (1 μg/ml, Thermo Fisher Scientific, Waltham, MA, USA) in PBS with 1 mg/ml BSA was added and incubated for 1 h. After washing with PBS-T, color was developed with TMB peroxidase substrate system (KPL, Gaithersburg, MD, USA) according to the manufacturer’s instructions.

After the screening experiment, the subsequent experiments were further optimized. The denatured Hb or HbA_1c_ was neutralized by the addition of more than ten times the initial volume of 0.1 M sodium carbonate buffer (pH 9.5) for the efficient binding to the ELISA plate. Furthermore, the plates were kept at 4 °C after the addition of biotinylated AAL for the reproducibility. The inhibitory effect of the anti-HbA_1c_ antibody (10 μg/ml) was determined by adding it to the neutralized HbA_1c_ after denaturation. For the assay of the inhibitory effect of sugars, biotinylated AAL was incubated with 10 mM l-fucose, d-fucose, d-fructose, or d-glucose before its addition to the plate. All data are shown as the mean value of at least three measurements with error bars of one standard deviation.

## Results

### Screening of HbA_1c_-binding lectins

**Fig. 1:**
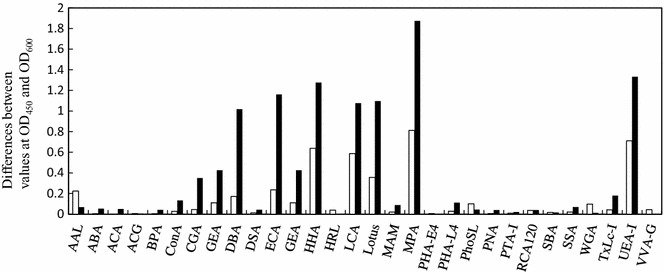
Screening of hemoglobin A_1c_-binding lectins. The denatured hemoglobin A_1c_ (*white*) or hemoglobin (*black*) was immobilized on the surface of the plate before the neutralization. The binding of biotinylated lectins was assayed by the activity of the specifically bound streptavidin-HRP

**Fig. 2:**
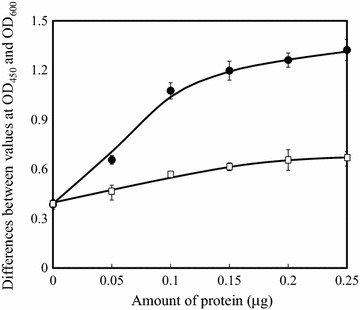
Binding between the lectin from *Aleuria aurantia* (AAL) and HbA_1c_ or Hb. The denatured HbA_1c_ (*filled circle*) or Hb (*open square*) were immobilized on the surface of the plate and the amount of biotinylated AAL bound to the protein was assayed by the activity of the specifically bound streptavidin-HRP

HbA_1c_-binding lectins were screened from 30 sources using lectin-based ELISA, as described in the “Materials and methods” section. Among the screened lectins, eight (AAL, DBA, ECA, HHA, LCA, Lotus, MPA, and UEA-I) were found to bind to HbA_1c_, although they showed similar or higher binding-affinity to Hb except for AAL (Fig. 1). AAL was selected for further experiments as it was found to bind with HbA_1c_, but not with Hb. The binding Fig. 2 shows the binding between AAL and HbA_1c_ and between AAL and Hb. The results show that AAL binding increased as the HbA_1c_ concentration increased. However, only a small increase in binding was observed when the Hb concentration increased. The neutralization before the addition of the ELISA plates increased the values of ELISA, because high pH buffer can increase the solubility of the proteins and makes the proteins unprotonated which helps binding to a positively charged ELISA plates.

### Effect of temperature

Binding affinities between lectins and saccharides are often enhanced at lower temperatures. The effect of temperature on the lectin-based ELISA was determined by decreasing the solution temperature after the addition of biotinylated AAL as described in the “Materials and methods” section. Although the solution temperature has a slight effect on the average values of binding to HbA_1c_ (data not shown), higher reproducibility was observed at lower temperatures. The average of coefficient of validation for the values of HbA_1c_ binding, as shown in Fig. 2, was 0.049. However, this average value at 25 and 37 °C, increased to 0.080 and 0.11, respectively (data not shown).

### Inhibitory effect of the anti-HbA_1c_ antibody

**Fig. 3:**
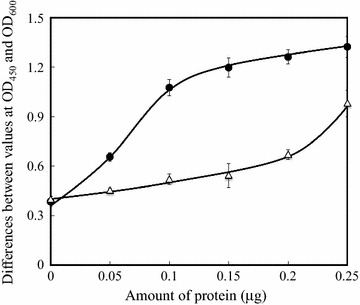
Inhibition of the binding by the monoclonal antibody against HbA_1c_. The lectin-based ELISA was performed in the presence (*open triangle*) and the absence (*closed circle*) of the anti-HbA_1c_ monoclonal antibody (10 μg/ml)

The inhibitory effect of the monoclonal antibody against the *N*-terminal fructosyl valine of β-chain of HbA_1c_ on the binding between AAL and HbA_1c_ was assayed in order to obtain information about the binding position of HbA_1c_. The antibody was incubated with denatured HbA_1c_ and lectin-based ELISA was performed. Figure 3 shows the effect of the antibody on the binding. The binding was inhibited by the incubation of the antibody and HbA_1c_ prior to the lectin-based ELISA. This result suggests that AAL binds to the *N*-terminal fructosyl valine of the β-chain of HbA_1c_.

l-Fucose occupies the binding sites of AAL and inhibits the binding of AAL to l-fucose-containing glycans. The binding sites of AAL to HbA_1c_ were assumed by determining the inhibition effects of various sugars. Table 1 shows the effect of 10 mM l-fucose, d-fucose, d-fructose, d-glucose, and d-arabinose on the interaction between AAL and HbA_1c_. The inhibition by d-fructose was higher than that by the other sugars, including l-fucose. These results suggest that AAL binds to the *N*-terminal fructosyl valine at a binding site distinct from that of l-fucose.

**Table 1:** Inhibitory effect of sugars on the values of the lectin-based ELISA

	Values^a^	Inhibition (%)
None	0.81	0
l-Fucose	0.60	26
d-Fucose	0.70	14
d-Fructose	0.47	42
d-Glucose	0.68	16
d-Arabinose	0.67	17

^a^The values of the lectin-based ELISA for 0.15 μg Hb_A1c_ in the presence and the absence of 10 mM sugars. The control values are reduced

## Discussion

**Fig. 4:**
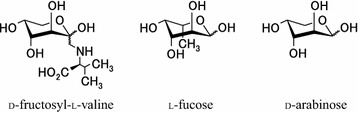
Comparison of structural formulas of fructosyl valine and the known ligands of AAL

This report revealed that AAL binds to HbA_1c_, which is a glucose modified Hb. This is the first report showing interaction between HbA_1c_ and AAL, which has been shown to interact with saccharides containing l-fucose or d-arabinose. The results of the lectin-based ELISA (Fig. 2) showed that AAL has more binding affinity with HbA_1c_ than that with Hb. Since the modification by glucose differentiates HbA_1c_ from Hb, AAL is suggested to bind to fructosyl valine of the β-chain of HbA_1c_. This hypothesis is consistent with the observed inhibitory effect of the antibody against fructosyl valine (Fig. 3). This binding is explained by the broad binding affinity of lectin. AAL also binds to d-arabinose, which lacks the C-6 methyl group of l-fucose, although the binding affinity is 30 times weaker than that to l-fucose (Fujihashi et al. 2003). Figure 4 compares the structural formulas of the hypothetical ligand d-fructosyl valine and its known ligands l-fucose and d-arabinose. Three of these pyranoses share the same configuration of all four hydroxyl groups at chiral carbons (C-1, C-2, C-3, and C-4). It is possible that AAL binds to the fructosyl valine of β-chain of HbA_1c_, as does l-fucose-containing saccharides. However, the binding may be partially distinct from each other, which could result in the distinct inhibition in the presence of l-fucose or l-fructose.

The reproducibility increased at 4 °C as compared to 25 and 37 °C. These results are likely caused by the higher binding affinity at lower temperatures. Higher affinity at lower temperature is often reported in the interactions between lectins and saccharides. Damian et al. (2005) suggested that the higher affinity at lower temperatures is caused by stabilization of water molecules that contribute to the binding. The interaction between AAL and HbA_1c_ may require water molecules.

AAL could be used for the assay of HbA_1c_ in human blood for the diagnosis of diabetes. Since AAL can be overproduced in *E. coli* and shows higher thermostability, a diagnostic test using AAL may be superior to the conventional assay using the antibody against HbA_1c_.

## Abbreviations

HbA_1c_: hemoglobin A_1c_
Hb: hemoglobin
AAL: lectin from *Aleuria aurantia*
ELISA: enzyme-linked immunosorbent assay
DBA: lectin from *Dolichos biflorus*
ECA: lectin from *Erythrina cristagalli*
LCA: lectin from *Lens culinaris*
Lotus: lectin from *Lotus tetragonolobus*
HHA: lectin from *Hippeatrum hybrid*
MPA: lectin from *Maclura pomifera*
UEA-1: lectin from *Ulex europaeus*
